# Retroperitoneal Necrotizing Fasciitis from Fournier's Gangrene in an Immunocompromised Patient

**DOI:** 10.1155/2017/5290793

**Published:** 2017-12-03

**Authors:** Samuel B. Weimer, Marc R. Matthews, Daniel M. Caruso, Kevin N. Foster

**Affiliations:** ^1^Department of Surgery, Maricopa Integrated Health Systems, Phoenix, AZ, USA; ^2^The Arizona Burn Center, Maricopa Integrated Health System, Phoenix, AZ, USA

## Abstract

**Introduction:**

Necrotizing fasciitis (NF) is a devastating soft tissue disease causing fulminant clinical deterioration, and extension into the retroperitoneum has a high mortality rate. This disease process demands a strong clinical suspicion for early identification which must be coupled with frequent wide surgical debridements and intravenous antibiotics for improved outcomes. Various clinical risk factors may render a weakness in the patient's immune status including diabetes mellitus, chronic renal failure, obesity, and autoimmune disorders, such as a human immunodeficiency virus (HIV) infection.

**Case Report:**

A 55-year-old male presented with hypotension requiring a large intravenous fluid resuscitation and vasopressors. He was diagnosed with the human immunodeficiency virus upon presentation. A computerized tomographic scan revealed air and fluid in the perineum and pelvis, ascending into the retroperitoneum. Multiple surgical debridements to his perineum, deep pelvic structures, and retroperitoneum were completed. After colostomy placement, antibiotic administration, and wound care, he was closed using split-thickness skin grafting.

**Conclusion:**

NF is a sinister and fulminant disease requiring prompt diagnosis and surgical intervention. The best chance for survival occurs with emergent surgical debridement and appropriate intravenous antibiotics. While retroperitoneal NF is consistent with uniformly poor outcomes, patients are best treated in an American Burn Association-verified burn center.

## 1. Introduction

Necrotizing fasciitis (NF) of the perineum, groin, and genitalia (historically referred to as Fournier's Gangrene (FG) [1]) is a devastating, soft tissue disease causing fulminant clinical deterioration in any patient regardless of the patient's immune status [2]. The presence of cellulitis occurs over infected subcutaneous tissue and fascia and eventually may lead to necrosis of the skin and muscles. NF rapidly expands along fascial planes and can further expand into the thighs, buttocks, and anterior abdominal wall [1–5]. To provide the greatest possibility for survival, it is vital to expeditiously identify the presence of NF. In NF's most severe form, patients present with pain out of proportion to the examination, subcutaneous emphysema, erythema, and necrosis at the site of the cutaneous infection [2]. Abnormal vital signs may include fever, hypotension, tachycardia, and tachypnea [1–3, 6]. While more men are affected than women at a 3-to-1 ratio, overall NF mortality ranges from 8.7% to 73% [2, 3]. Various clinical risk factors that may render a weakness in a patient's immune status are frequently seen including diabetes mellitus, chronic renal failure, obesity, and autoimmune disorders, such as a human immunodeficiency virus (HIV) infection [2, 6]. A traumatic injury to the skin may be the inciting event leading to an inoculum of bacteria into the host's system. NF may harbor either aerobic or anaerobic, Gram-positive or -negative bacteria, or a synergistic combination of both, which may also produce gas, such as with *Clostridium perfringens* [4, 5].

If left untreated or inadequately debrided, NF may rapidly progress into septic shock which has a mortality rate that approaches 100% [1–3, 7–9]. This disease process mandates aggressive and extensive surgical debridement with frequently timed reexcisions for adequate source control coupled with systemic intravenous antibiotics for improved survival [3–5, 10]. However, antibiotics alone are not considered adequate therapy, nor will antibiotics cease NF's progression without wide and aggressive surgical debridement. Rarely does NF directly invade into the pelvis or retroperitoneum from the more superficial fascial levels along interfascial planes; however, if it does, NF is even more difficult to manage because of its presence within the deep pelvis and retroperitoneum of the torso [10–13].

There is a paucity of reports that have been published regarding retroperitoneal necrotizing fasciitis and even fewer that have shown successful clinical outcomes [6, 8] after treatment for this fulminant disease process, especially in an immunocompromised patient with HIV [7]. Presented here is the successful treatment of a 55-year-old, newly diagnosed, HIV-positive male with necrotizing fasciitis of the perineum and testicles that spreads into the retroperitoneum after invasion through the pelvis requiring multiple operations and wound therapy.

## 2. Case Report

A 55-year-old male presented to the emergency department (ED) with a several day history of nausea, vomiting, and diarrhea. The patient was hypotensive with a blood pressure (BP) of 84/63 mmHg, a heart rate of 123 beats/min, and a temperature of 37.1 Celsius. On laboratory examination, he had a white blood cell count of 12 mm^3^, a lactate level of 8.1 mmol/L, a sodium level of 129 mEq/L, and a total bilirubin level of 2.4 mg/dL. His creatinine level of 2.3 mg/dL was consistent with acute kidney injury. Due to emergency department screening protocols, the patient was tested and found to be positive for the human immunodeficiency virus (HIV), which he was unaware of at the time of his presentation. The patient was aggressively resuscitated with three liters of intravenous crystalloid with a moderate improvement in his systolic blood pressure.

**Figure 1:**
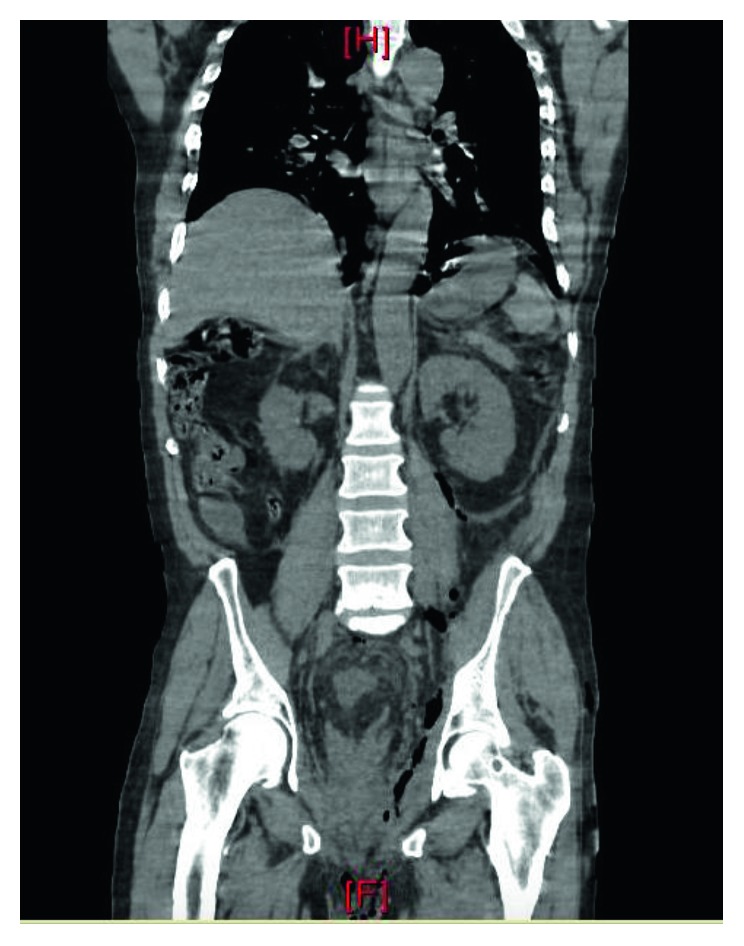
Coronal CT image showing gas tracking from left perirectal area superiorly into the left retroperitoneum to the level of the medial and inferior left kidney.

**Figure 2:**
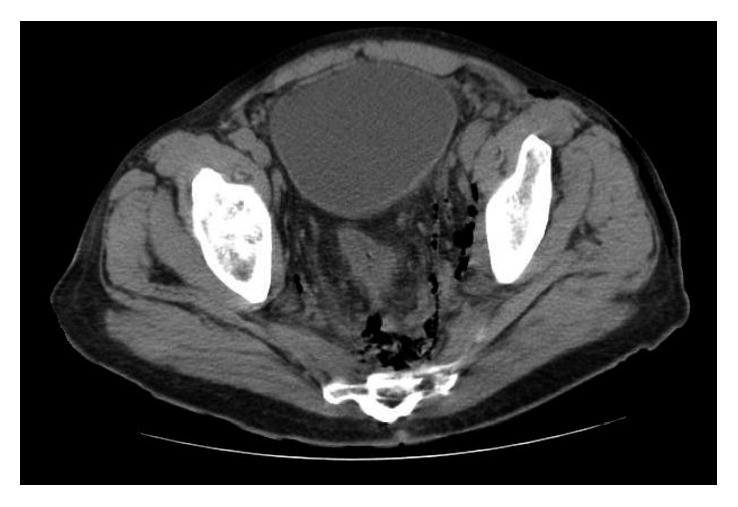
Axial CT image showing gas around the left rectum to the sacrum and ascending anterior along the left internal pelvic sidewall.

**Figure 3:**
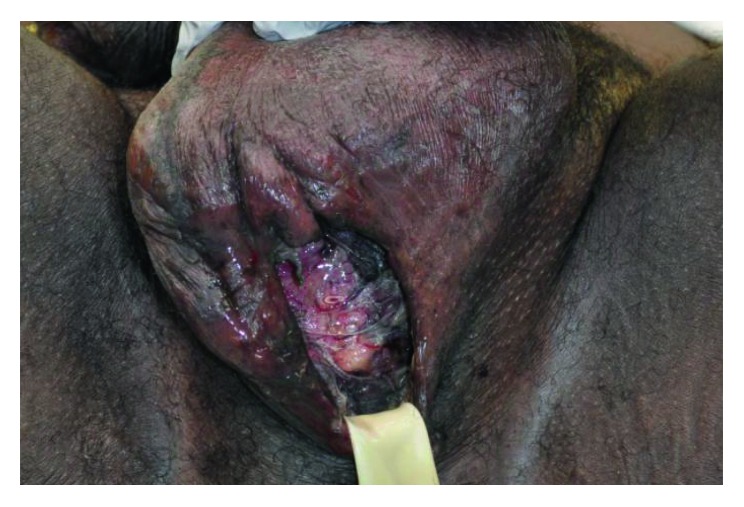
Photographic image of the necrotic tissue of the scrotum with a Penrose drain inserted after an inadequate debridement.

**Figure 4:**
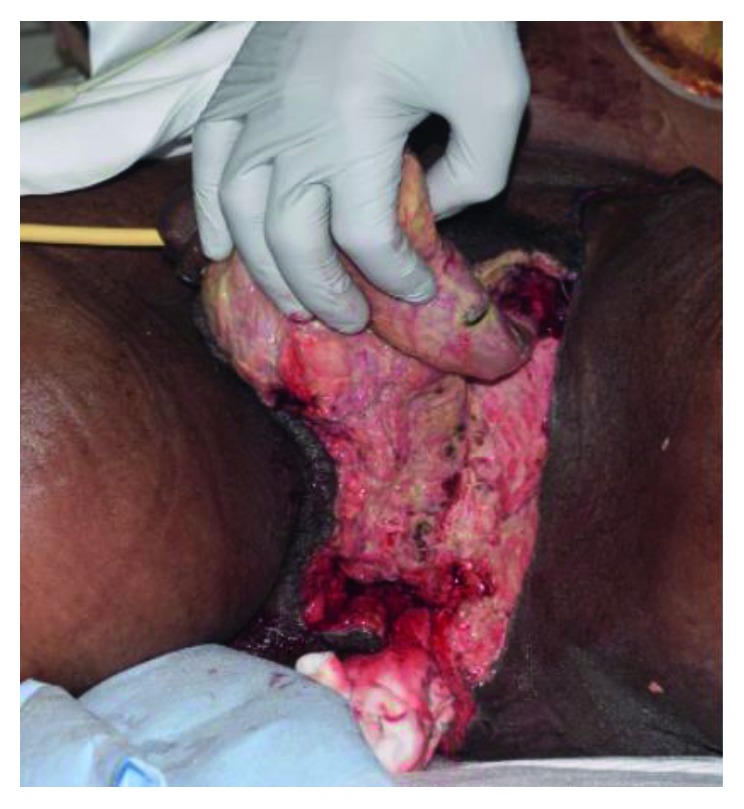
Photographic image of the perineum and scrotum post debridement of involved skin and subcutaneous tissue and packing at the perineal level extending into the pelvis.

**Figure 5:**
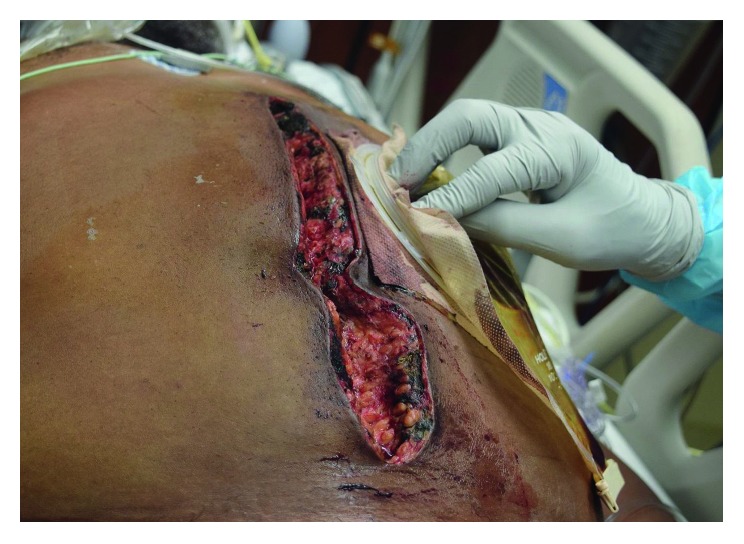
Photographic image of the patient's midline abdominal closure and colostomy bag after left-sided colostomy creation for fecal diversion.

He was admitted to the medical intensive care unit (ICU) for continued resuscitation and further evaluation of the persistent hypotension and tachycardia. Blood cultures were obtained, and empiric intravenous antibiotics were initiated, as well as intravenous vasopressor support (norepinephrine (Hospira, Inc., Lake Forest, IL)) to maintain a mean arterial pressure (MAP) greater than 65 mmHg. A computerized tomography (CT) scan of the abdomen and pelvis revealed significant air, fluid, and inflammation around the rectum and scrotum tracking proximally through the pelvis and into the left retroperitoneum with inflammation just below the left kidney (Figures 1 and 2) which prompted a surgical consultation. The patient underwent emergent debridement of the perineal and scrotal abscesses and was admitted to the American Burn Association- (ABA-) verified burn center (Figure 3). The patient was repeatedly operated upon every 24 hours for a more extensive perineal and scrotal debridement (Figure 4). On early hospital day three, the patient underwent an exploratory laparotomy for a left colonic medial visceral rotation with extensive debridement of the left retroperitoneum. Infected necrotic tissue and foul-smelling exudate were found in the retroperitoneum requiring a wide excision of the left psoas muscle, lateral abdominal wall, and Gerota's fascia of the left kidney and ligation of a thrombosed left gonadal vein. Anoscopy was performed, which identified a left posterior fistula, communicating with the left ischiorectal space, which was identified to be the initiating site of this soft tissue necrotizing infection. A temporary negative pressure wound therapy device (temporary abdominal closure (TAC) device) was placed to the open abdomen, and the patient returned to the OR daily for further abdominal washouts and retroperitoneal debridements until the infection had been completely debrided. The anal sphincter complex required complete excision secondary to necrosis which then necessitated a proximal colonic diversion for control of his fecal incontinence (Figure 5).

Tissue cultures revealed a polymicrobial mixture of *Parabacteroides distasonis*, *Prevotella melaninogenica*, and *Fusobacterium nucleatum*, while his blood cultures grew *Bacteroides thetaiotaomicron*. He was treated with intravenous piperacillin/tazobactam (Sandoz, Princeton, NJ) and linezolid (Pfizer, Inc., New York, NY). Initially, the patient was noted to have a CD4 count of 14 and a viral load greater than 349,000. His chest X-ray (CXR) revealed a diffuse interstitial prominence and peribronchial cuffing while sputum studies revealed a *Pneumocystis jirovecii* infection. The patient was treated with clindamycin (ACS Dobfar SPA, Switzerland) and primaquine (Sanofi-Aventis, St. Louis, MO) for approximately 3 weeks followed by Mepron (GlaxoSmithKline, Research Triangle Park, NC) for prophylaxis. Antiretroviral therapy was initially deferred due to the patient's hemodynamic instability and renal failure; however, Triumeq (ViiV, Brentford, UK) was eventually initiated, once his hemodynamic condition stabilized.

**Figure 6:**
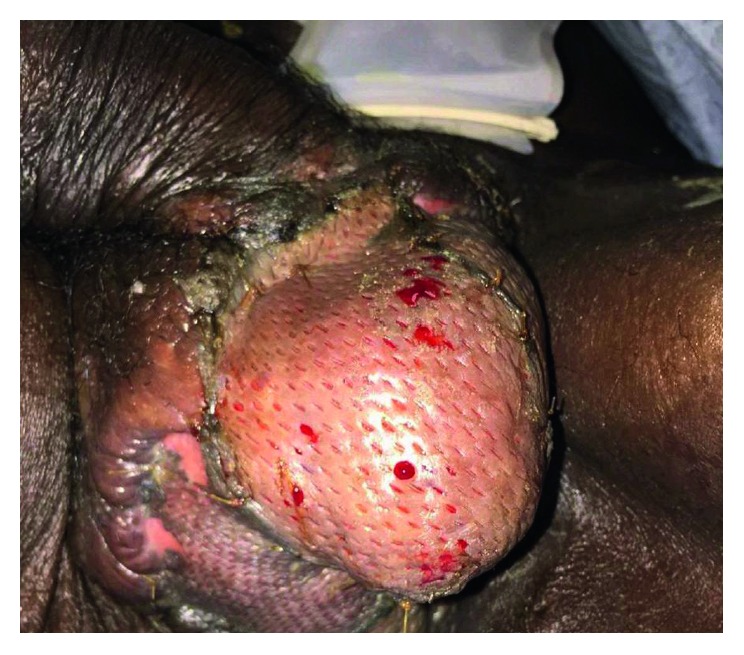
Photographic image of the patient's reconstruction using 1-to-1 meshed split-thickness skin grafts to the testicles to create a neoscrotum.

The patient spent three months admitted to the burn center and was subsequently discharged to a skilled nursing facility with follow-up in the burn clinic. After approximately six weeks convalescing, there was sufficient granulation tissue on the perineum and testicles, and he underwent one-to-one meshed split-thickness skin grafting for cutaneous coverage and creation of a neoscrotum for the testicles (Figure 6). The patient was discharged by the fifth postoperative day with follow-up in the burn center clinic.

## 3. Discussion

Necrotizing fasciitis is a fulminant, soft tissue necrotizing disease occurring in any region of the body and has a high mortality rate especially if the diagnosis is inaccurate or delayed [1]. Early and accurate diagnosis of this disease is of utmost importance and requires prompt surgical evaluation and aggressive debridement [3, 5]. One study identified that delay in debridement greater than 24 hours from initial diagnosis had a significant increase in mortality [3]. A potential delay in surgical intervention, with initial admission to a medical service instead of a surgical service, also resulted in increased mortality [5]. If the NF involves the perineum, testicles, or groin as in FG and progresses into the pelvis, access to the internal pelvis from the perineum by the surgeon can be challenging especially in the well-vascularized, deep recesses where there is the threat of inadvertent arterial or venous injury with concomitant hemorrhage. This may possibly require a ventral approach through the anterior midline with more proximal control at the common aortoiliac arterial or iliocaval level. Retroperitoneal NF also requires an abdominal exploration with a colonic medial visceral rotation to gain access of the posterior abdominal wall musculature or organs for adequate debridement. Abdominal wall muscle debridements are possible until the necrotic tissue removal involves the erector spinae muscles. Erector spinae muscle debridement may result in spine instability, loss of major nerves emanating from the vertebral foramina with subsequent loss of distal nervous function, or infectious exposure into the spinal canal. Perineal dressing changes with internal pelvic packing become an every six-to-twelve hour necessity performed by the ICU staff. Retroperitoneal NF may require leaving the abdomen open with a temporary abdominal closure (TAC) device, all of which can be extremely taxing for the patient and adding further burden to the resources involved in the ICU care. Irrigating negative pressure wound therapy applied to the ventral open abdomen or large Davol drains (Davol, a Bard Company, Warwick, RI) placed in the retroperitoneum can be of benefit by lavaging the exposed, septic retroperitoneum.

Computerized tomographic imaging is generally obtained to establish the broad extent and depth of the NF process within that specified body region especially for the retroperitoneum [14]. While plain radiography can be obtained revealing gas formation in the soft tissues, it is less sensitive and specific, while magnetic resonance imaging is too costly despite providing better accuracy [2]. In Woodburn's case series, 100% of retroperitoneal NF patients died; nonetheless, CT scans provided the best visualization of the retroperitoneal infectious process even though such changes were suggestive of advanced and preterminal disease [8]. The temporary use of CT-guided drain placement has also been suggested [14]; however, it does not negate the need for adequate surgical debridement and, secondarily, intravenous antibiotics.

The diagnosis of NF relies on clinical findings which can only be supplemented by clinical and laboratory scores such as the Laboratory Risk Indicator for Necrotizing Fasciitis (LRINEC) score [15–17] or the Fournier's Gangrene Severity Index [18]. However, the use of the LRINEC score has been called into question [17, 19, 20] and does not supplant clinical evaluation and mandatory surgical assessment [14]. If clinically unsure of the diagnosis, a surgical incision over the fascia at the area of concern may be performed under local, regional, or general anesthesia to look for signs of necrotizing fasciitis [3]. Classic intraoperative findings include “dishwater fluid” exudate and necrotic or nonviable tissue that does not bleed upon incision. In addition, subcutaneous tissue planes are easily separated away from the fascia or necrotic fascia and muscles [2, 7]. Once confirmed, wide excision of all necrotic tissue is imperative to control the necrotizing process. As a general guide, the tissue must be excised to healthy, bleeding tissue and continuously reevaluated in the OR every 24 hours until source control is achieved [21]. It is clear that the admonition as suggested by Burge and Watson, as well as others, who cited William Shakespeare from Macbeth, Act 4, Scene 1, in that achieving adequate surgical margins when excising NF should be “bloody, bold and resolute” is absolutely imperative [5, 22–24]. Between operative debridements, topical Dakin's solution (sodium hypochlorite) (Century Pharmaceuticals, Indianapolis, IN) or Vashe solution (hypochlorous acid/HOCl) (Steadmed, Fort Worth, TX) dressings have been used to treat the open wounds and have proven to be effective against a broad spectrum of bacteria in the patient's wound beds [10, 25–27].

In regard to hyperbaric oxygen (HBO) administration for the patient with necrotizing fasciitis, there are many benefits, including, but not limited to, a direct anaerobic bacterial effect especially for clostridial species, a decrease in endotoxin activity, an increase in leukocytic phagocytosis, and a reduction in free radical production. However, the downside risk to placing a patient in a HBO chamber includes, first, the loss of medical control of the patient especially if the HBO chamber is not located on that medical center's campus where the patient would have to travel to an off-site location and, second, a delay in the mandatory and repeated surgical debridement while the patient received such HBO therapy. The trade-offs between risk versus benefit must be weighed very carefully in these extremely sick patients; nonetheless, if managed appropriately, HBO therapy is a useful clinical adjunct in our opinion.

For this patient, his unknown and newly diagnosed HIV status increased his risk of morbidity and mortality and complicated his overall care [8, 11]. *Pneumocystis jirovecii* pneumonia is considered an autoimmune deficiency syndrome (AIDS) defining illness as well as his low CD4 count. In another similar case report, a HIV-positive male with a severe retroperitoneal soft tissue infection had no leukocytosis on admission which parallels our patient's course [6]. Unfortunately, the lack of leukocytosis delayed a sepsis workup and ultimately led to the patient's admission to a nonsurgical service and a delay in surgical intervention. It is also important that NF be managed in a facility that has the expertise and resources to appropriately manage the acute wound care and reconstructive needs such as an ABA-verified burn center [4, 5]. Two previous studies have noted a decrease in mortality with early transfer to a burn care facility [4, 5]. These patients require expertise in surgical critical care and operative management, complicated wound-dressing management, and definitive surgical reconstruction since burn injuries and NF are treated similarly.

## 4. Conclusion

NF is a sinister and fulminant disease requiring prompt diagnosis and surgical intervention especially with its presence in the deep pelvis or the retroperitoneum in a newly diagnosed HIV-positive patient. The best chance for survival with a return to a good quality of life occurs with a high index of suspicion and emergent surgical debridement and appropriate antibiotics when managed by a surgical critical care team capable of handling such complicated cases. While retroperitoneal NF is consistent with uniformly poor outcomes, patients are best treated with admission to an intensive care unit within an ABA-verified burn center.
